# Muscle Glycogen Depletion Following 75-km of Cycling Is Not Linked to Increased Muscle IL-6, IL-8, and MCP-1 mRNA Expression and Protein Content

**DOI:** 10.3389/fphys.2016.00431

**Published:** 2016-09-27

**Authors:** David C. Nieman, Kevin A. Zwetsloot, Dominic D. Lomiwes, Mary P. Meaney, Roger D. Hurst

**Affiliations:** ^1^Appalachian State University, North Carolina Research CampusKannapolis, NC, USA; ^2^The New Zealand Institute for Plant and Food Research Ltd.Palmerston North, New Zealand

**Keywords:** exercise, inflammation, muscle damage, stress hormones, chemokines

## Abstract

The cytokine response to heavy exertion varies widely for unknown reasons, and this study evaluated the relative importance of glycogen depletion, muscle damage, and stress hormone changes on blood and muscle cytokine measures. Cyclists (*N* = 20) participated in a 75-km cycling time trial (168 ± 26.0 min), with blood and vastus lateralis muscle samples collected before and after. Muscle glycogen decreased 77.2 ± 17.4%, muscle IL-6, IL-8, and MCP-1 mRNA increased 18.5 ± 2.8−, 45.3 ± 7.8−, and 8.25 ± 1.75-fold, and muscle IL-6, IL-8, and MCP-1 protein increased 70.5 ± 14.1%, 347 ± 68.1%, and 148 ± 21.3%, respectively (all, *P* < 0.001). Serum myoglobin and cortisol increased 32.1 ± 3.3 to 242 ± 48.3 mg/mL, and 295 ± 27.6 to 784 ± 63.5 nmol/L, respectively (both *P* < 0.001). Plasma IL-6, IL-8, and MCP-1 increased 0.42 ± 0.07 to 18.5 ± 3.8, 4.07 ± 0.37 to 17.0 ± 1.8, and 96.5 ± 3.7 to 240 ± 21.6 pg/mL, respectively (all *P* < 0.001). Increases in muscle IL-6, IL-8, and MCP-1 mRNA were unrelated to any of the outcome measures. Muscle glycogen depletion was related to change in plasma IL-6 (*r* = 0.462, *P* = 0.040), with change in myoglobin related to plasma IL-8 (*r* = 0.582, *P* = 0.007) and plasma MCP-1 (*r* = 0.457, *P* = 0.043), and muscle MCP-1 protein (*r* = 0.588, *P* = 0.017); cortisol was related to plasma IL-8 (*r* = 0.613, *P* = 0.004), muscle IL-8 protein (*r* = 0.681, *P* = 0.004), and plasma MCP-1 (*r* = 0.442, *P* = 0.050). In summary, this study showed that muscle IL-6, IL-8, and MCP-1 mRNA expression after 75-km cycling was unrelated to glycogen depletion and muscle damage, with change in muscle glycogen related to plasma IL-6, and changes in serum myoglobin and cortisol related to the chemotactic cytokines IL-8 and MCP-1.

## Introduction

The cytokine response across a range of exercise workloads has been broadly described (Peake et al., 2015). In general, intensive and sustained exercise bouts result in high plasma cytokine concentrations when compared to more moderate activity, with the greatest fold changes measured for IL-6, IL-8, IL-10, and granulocyte colony stimulating factor (G-CSF), interleukin-1 receptor antagonist (IL-1ra), and monocyte chemoattractant protein-1 (MCP-1) (Nieman et al., 2003, 2005, 2006, 2007, 2012, 2014). Cytokine release during exercise assists in many important physiological roles including control of inflammation and immune cell movement, enhancement of lipolysis and insulin sensitivity, and regulation of tissue repair and training adaptations (Pedersen, 2013; Catoire and Kersten, 2015; Peake et al., 2015).

The plasma cytokine response to prolonged and intensive exercise varies widely between individuals, and the relative importance of factors that explain this variance is still being investigated (Peake et al., 2015). These may include pre-exercise muscle glycogen levels and the rate of glycogen depletion during exercise, the extent of muscle damage, the exercise mode and workload, plasma stress hormone levels, medication use, carbohydrate intake and nutritional status, environmental stress, age, gender, and training status (Keller et al., 2001, 2003, 2005; Nieman et al., 2003, 2004, 2005, 2006, 2009, 2012, 2014, 2015; Chan et al., 2004; Holmes et al., 2004; Helge et al., 2011; Reihmane et al., 2013; Della Gatta et al., 2014; Wright et al., 2014).

Exercising with low muscle glycogen content has been related to increased muscle IL-6 and IL-8 mRNA expression and release (Keller et al., 2001; Steensberg et al., 2001; Chan et al., 2004). Athletes vary widely in glycogen depletion rates during prolonged and extensive exercise, and these studies imply that those with the highest rates may experience the greatest muscle cytokine mRNA expression. However, most investigators have not been able to confirm this proposed relationship (Holmes et al., 2004; Keller et al., 2005; Gusba et al., 2008; Helge et al., 2011; Nieman et al., 2015). Our research group recently reported that post-run muscle glycogen content was negatively related to plasma but not muscle mRNA levels for IL-6, IL-8, and MCP-1 in 24 male endurance athletes who ran to exhaustion on treadmills at ~70% VO_2max_ (Nieman et al., 2015). Additionally, no significant correlations were found between exercise-induced changes in stress hormones and plasma or muscle cytokines (both mRNA and protein levels). Limitations in this study included the lack of measurement of muscle damage, and a modest decrease in muscle glycogen sampled from the runner's vastus lateralis (35%).

The purpose of this study was to correlate changes in glycogen depletion (primary outcome), muscle damage, and the stress hormone cortisol following 75-km of cycling with changes in plasma cytokines, and muscle cytokine mRNA and protein levels in 20 trained cyclists. Muscle samples were biopsied from the vastus lateralis, a muscle that is more directly involved in generating power during long endurance cycling compared to running, and becomes more depleted in glycogen (Nieman et al., 2003, 2005, 2015). We hypothesized that significant, negative relationships would be measured between variance in vastus lateralis glycogen depletion and muscle IL-6, IL-8, and MCP-1 mRNA expression, change in muscle cytokine protein concentrations, and increases in plasma cytokine levels in cyclists after a 75-km cycling time trial.

## Methods

### Participants and baseline testing

Participant recruitment was conducted via direct messaging to cyclists and cycling clubs in the Charlotte, NC, metropolitan area. Participants included 20 male cyclists (ages 29–48 years) who regularly competed in road races and had experience with long distance cycling time trials. Participants voluntarily provided informed consent and all study procedures were approved by the Institutional Review Board at Appalachian State University.

One week prior to the 75-km time trial, each athlete completed orientation/baseline testing. Demographic and training histories were acquired with questionnaires. Maximal power, oxygen consumption, ventilation, and heart rate were measured during a graded exercise test (25 W increase every 2 min, starting at 150 W) with the Cosmed Quark CPET metabolic cart (Rome, Italy) and the Lode cycle ergometer (Lode Excaliber Sport, Lode B.V., Groningen, Netherlands). Body composition (whole body fat percentage through air displacement plethysmography technology) was measured with the Bod Pod body composition analyzer (Life Measurement, Concord, CA).

### Seventy five-kilometer cycling time trial

One week following baseline testing, participants completed in a 75-km cycling time trial on their own bicycles mounted on CompuTrainer Pro Model 8001 trainers (RacerMate, Seattle, WA). A mountainous 75-km course with moderate difficulty was chosen and programmed into the software system. Heart rate and rating of perceived exertion (RPE) were recorded at 15 min, and every 60 min thereafter, and workload in watts was continuously monitored using the CompuTrainer MultiRider software system (version 3.0). Oxygen consumption and ventilation were measured using the Cosmed Quark CPET metabolic cart (Rome, Italy) after 16 and 55 km cycling. Participants were allowed to ingest water *ad libitum* during the 75-km cycling time trial, but intake of other beverages and food were prohibited.

### Blood sample analysis

Blood samples were collected 30 min pre- and immediately post-exercise and analyzed for plasma glucose, plasma lactate, serum cortisol, serum myoglobin, and plasma cytokines. Plasma glucose and lactate were analyzed using the YSI 2300 STAT Plus Glucose and Lactate analyzer (Yellow Springs, OH).

Serum myoglobin was measured using an LX-20 clinical analyzer (Beckman Coulter Electronics, Brea, CA). Serum cortisol was analyzed with an electrochemiluminescence immunoassay (ECLIA) through a commercial lab (LabCorp, Burlington, NC). Total plasma concentrations of three cytokines [monocyte chemoattractant protein-1 (MCP-1), IL-6, and IL-8] were determined using an electrochemiluminescence based solid-phase sandwich immunoassay (Meso Scale Discovery, Gaithersburg, MD, USA). All samples and provided standards were analyzed in duplicate, and the intra-assay CV ranged from 1.7 to 7.5% and the inter-assay CV 2.4 to 9.6% for all cytokines measured. Pre-and post-exercise samples for the cytokines were analyzed on the same assay plate to minimize inter-kit assay variability.

### Muscle biopsy procedures

Muscle biopsy samples were acquired 30 min pre-exercise and immediately post-exercise on the same leg at the same vastus lateralis locations (2 cm apart). Local anesthesia (1% xylocaine, Hospira, Inc., Lake Forest, IL) was injected subcutaneously. After a small incision, a muscle biopsy sample was obtained using the suction-modified percutaneous needle biopsy procedure (Shanely et al., 2014). Muscle was trimmed of connective tissue and fat and immediately frozen in liquid nitrogen. Samples were stored at −80°C until subsequent analysis. A glycogen assay kit (Catalog #MAK016, Sigma-Aldrich, St. Louis, MO) was used to determine the concentration of glycogen in vastus lateralis muscle homogenates. In this coupled enzyme assay, glucoamylase hydrolyzed glycogen to glucose, and then the glucose was oxidized to yield a product that reacted with a probe to generate a color detectable with a microplate reader (Synergy H1 Hybrid Reader, BioTek Instruments, Inc., Winooski, VT) at 570 nm.

### Muscle mRNA measurements

Total RNA extraction of vastus lateralis biopsies was conducted using an RNeasy® Fibrous Tissue Mini Kit (Qiagen, Limburg, Netherlands) according to the manufacturer's instructions. Frozen muscle biopsies were homogenized in RTL –β-mercaptoethanol Buffer with a Precellys homogenizer (Bertin Technologies, Montigny-le-Bretonneux, France). An aliquot of RNA from all samples were run on 1.5 denaturing agarose gels stained with ethidium bromide to confirm RNA integrity. Additionally, RNA quality was further verified spectrophotometrically using the A_260_/A_280_ ratio which was determined using a nanodrop. Reverse transcription of RNA into cDNA was conducted using a High Capacity cDNA Reverse Transcription Kit (Applied Biosystems, Foster City, CA) as per manufacturer's instructions. Reverse transcription was performed using a GeneAmp® PCR System 9700 (Applied Biosystems). cDNA was quantified using a nanodrop and aliquots were stored at −20°C until Real Time-Polymerase Chain Reaction (RT-PCR) analysis.

Quantitative RT-PCR analysis of messenger RNA (mRNA) was conducted under pre-optimized TaqMan Gene Expression Assays (Applied Biosystems) using gene specific FAM dye-labeled primers for IL-6 (Cat. No. 4453320), IL-8 (Cat. No. 4369514), MCP-1 (Cat. No. 4331182) and beta-2-microglobulin (B2M) (Cat. No. 4351370). Samples and reagents were loaded in a MicroAmP Fast-Optical 96 Well Reaction Plate and run in quadruplicate. Plates were analyzed on a 7500 Fast RT-PCR System (Applied Biosystems). Relative mRNA levels were determined using the ΔΔC_t_ calculations with B2M serving as the housekeeping gene (Livak and Schmittgen, 2001). Changes in cytokine expression post exercise were normalized to pre-exercise levels.

### Analysis of skeletal muscle inflammatory cytokine protein concentrations

Skeletal muscle biopsy samples were analyzed for the inflammatory cytokines IL-6, IL-8, and MCP-1 with a magnetic bead-based multiplex assay using the MAGPIX instrument and xPONENT® analysis software (Luminex, Austin, TX). Briefly, ~30 mg of each skeletal muscle tissue biopsy sample was homogenized in lysis buffer (Millipore, Billerica, MA; #43-040) with AEBSF protease inhibitor added (Millipore; #101500). Homogenates were cleared by centrifugation at 14,000 x g and the protein concentration of the supernatant was determined using a BCA protein assay kit (Pierce ThermoFisher, Rockford, IL). Next, 30 μg of muscle protein was added to each well (in duplicate) and the concentration of each cytokine was measured using the MILLIPLEX® MAP assay kit (Millipore, #HCYTOMAG-60K) according to manufacturer's specifications. The lower limit of detection and inter-assay % coefficient of variability (% CV) for this panel of analytes was: IL-6 = 0.17 pg/mL (4.2% CV); IL-8 = 0.18 pg/mL (5.9% CV); and MCP-1 = 0.30 pg/mL (3.0% CV). The intra-assay %CVs were IL-6 = 13.6%, IL-8 = 8.9%, and MCP-1 = 3.7%.

### Statistical analysis

Data are expressed as mean ± *SD*. Pre- and post-exercise data were tested for change using paired *t*-tests, with Pearson correlations used to test relationships between outcome measures (alpha level *P* ≤ 0.05).

## Results

**Figure 1:**
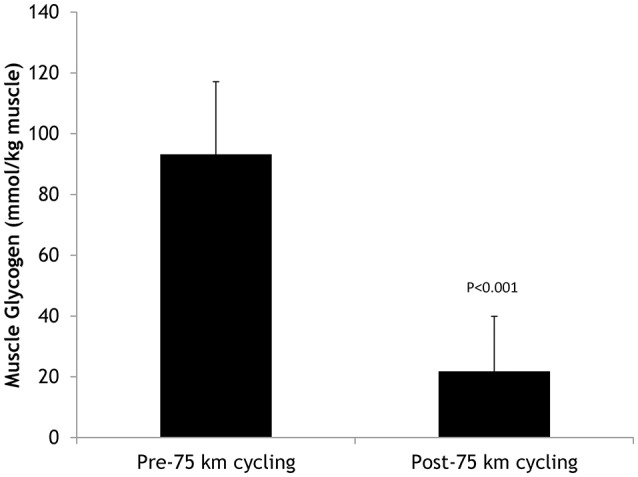
**Pre- and post-75-km cycling skeletal muscle glycogen levels in ***N*** = 20 cyclists**.

Table 1 summarizes participant characteristics for the 20 male cyclists (age range, 29–48 years). The cyclists completed the 75-km cycling time trial in 168 ± 26.0 min at a high metabolic intensity or 69.6 ± 10.3% VO_2max_ (Table 2). Participants reported an RPE of 12.4 ± 1.5 at 15 min, 13.1 ± 1.5 at 60 min, 14.6 ± 1.8 at 120 min, and 17.6 ± 0.7 (“very hard”) at the end of the 75-km cycling trial (average of 14.7 ± 1.6 for the entire time trial). Serum cortisol increased 196 ± 129%, serum myoglobin 794 ± 1025%, and plasma lactate 125 ± 29.3%, with no significant change in plasma glucose, by the end of the 75-km cycling time trial (Table 3). Muscle glycogen decreased 77.2 ± 17.4% (Figure 1), with an absolute change of 71.4 ± 23.1 (range 32–110) mmol glycogen per kilogram wet weight of muscle (*P* < 0.001).

**Table 1:** **Participant characteristics (***N*** = 20 male cyclists)**.

**Variable**	**Mean ±*SD***
Age	38.4 ± 6.0
Height (m)	1.82 ± 0.7
Weight (kg)	83.3 ± 7.2
Body fat (%)	20.3 ± 5.9
VO_2max_ (ml.kg^−1^min^−1^)	47.9 ± 7.8
Maximal heart rate (beats/min)	179 ± 8.6
Watts_max_	351 ± 57.6
Maximal ventilation (L/min)	128 ± 17.1
Maximal respiratory rate (breaths/min)	46.7 ± 7.4
Training (km/week)	154 ± 93.5

*SD, standard deviation; VO_2max_, maximal oxygen consumption*.

**Table 2:** **Performance variables averaged for entire 75 km cycling time trial (***N*** = 20 male cyclists)**.

**Variable**	**mean ± *SD***
VO_2_ (ml.kg^−1^min^−1^)	33.2 ± 6.4
%VO_2max_	69.6 ± 10.3
Watts	193 ± 57.8
%Watts_max_	54.2 ± 9.6
HR (beats/min)	160 ± 11.5
%HR_max_	89.4 ± 5.9
Ventilation (L/min)	74.0 ± 16.7
Rating Perceived Exertion (RPE)	14.7 ± 1.6

*SD, standard deviation; VO_2_, oxygen consumption; VO_2max_, maximal oxygen consumption; HR, heart rate*.

**Table 3:** **Cortisol, myoglobin, lactate, and glucose concentrations from blood samples in ***N*** = 20 male cyclists (mean ± ***SD***)**.

**Variable**	**Pre-75 km cycling**	**Post-75-km cycling**	***P*-value**
Serum cortisol (μg/dL)	10.7 ± 4.3	28.4 ± 10.5	<0.001
Serum myoglobin (ng/mL)	32.1 ± 14.7	242 ± 216	<0.001
Plasma lactate (mmol/L)	0.97 ± 0.3	2.02 ± 1.0	<0.001
Plasma glucose (mmol/L)	3.88 ± 0.78	4.25 ± 0.75	0.185

*P-value from paired t-test*.

**Figure 2:**
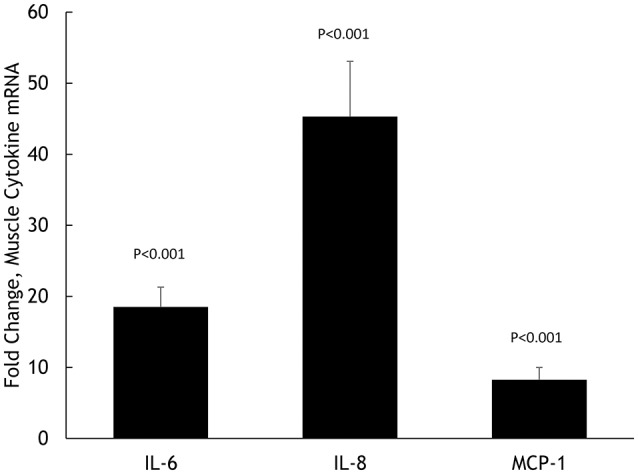
**Fold change in skeletal muscle mRNA expression for IL-6, IL-8, and MCP-1 in ***N*** = 20 cyclists in response to 75-km cycling**.

**Figure 3:**
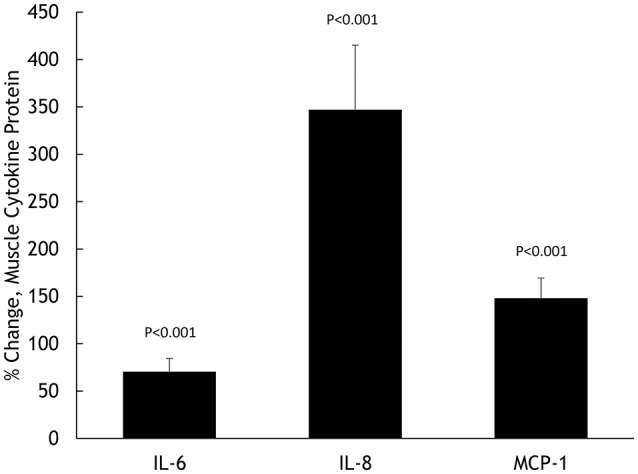
**Percent change in skeletal muscle cytokine protein content for IL-6, IL-8, and MCP-1**.

**Figure 4:**
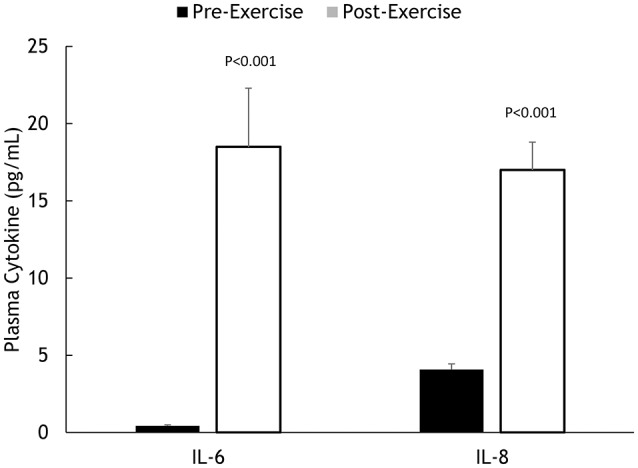
**Pre- and post-exercise plasma IL-6 and IL-8 concentrations**.

**Figure 5:**
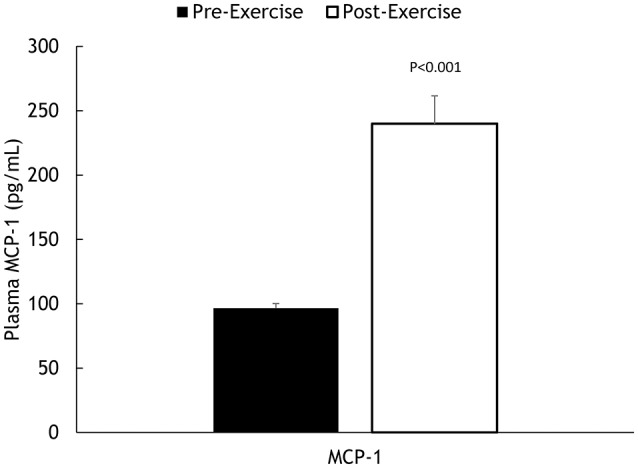
**Pre- and post-exercise plasma MCP-1 concentrations**.

Muscle IL-6, IL-8, and MCP-1 mRNA increased 18.5 ± 2.8-, 45.3 ± 7.8-, and 8.25 ± 1.75-fold, respectively, all *P* < 0.001 (Figure 2). Muscle IL-6, IL-8, and MCP-1 protein increased 70.5 ± 14.1%, 347 ± 68.1%, and 148 ± 21.3%, respectively, all *P* < 0.001 (Figure 3). Plasma IL-6, IL-8, and MCP-1 increased from 0.42 ± 0.07 to 18.5 ± 3.8, 4.07 ± 0.37 to 17.0 ± 1.8, and 96.5 ± 3.7 to 240 ± 21.6 pg/mL, respectively, all *P* < 0.001 (Figures 4, 5).

Performance measures (time, %watts_max_), and changes in muscle glycogen, serum myoglobin, serum cortisol, and plasma IL-6, IL-8, and MCP-1 were unrelated to change in muscle IL-6, IL-8, and MCP-1 mRNA. Muscle glycogen change was related to change in plasma IL-6 (*r* = −0.462, *P* = 0.040), with relationships found for serum myoglobin with plasma IL-8 (*r* = 0.582, *P* = 0.007) and plasma MCP-1 (*r* = 0.457, *P* = 0.043), and muscle MCP-1 protein (*r* = 0.588, *P* = 0.017). Serum cortisol was related to plasma IL-8 (*r* = 0.613, *P* = 0.004) and plasma MCP-1 (*r* = 0.442, *P* = 0.050), and muscle IL-8 protein (*r* = 0.681, *P* = 0.004).

## Discussion

Twenty well-trained male cyclists engaged in a 75-km cycling time trial and experienced an average decrease of approximately three-fourths of glycogen content in the vastus lateralis, and significant increases in the muscle damage biomarker serum myoglobin (794%) and the stress hormone serum cortisol (196%). Large post-exercise increases were measured in muscle IL-6, IL-8, and MCP-1 mRNA expression and protein levels, and plasma levels of these three cytokines. As expected, the decrease in vastus lateralis glycogen content following 75-km cycling was more than twice as great as measured following treadmill running to exhaustion (Nieman et al., 2015).

Pre-to-post-exercise changes in all of these cytokine measures varied widely between study participants as indicated by the large standard deviations. For example, the increase in plasma IL-6 varied from 2.7 to 73.6 pg/mL, with fold change for IL-6 mRNA varying from 3.1 to 42.6. This purpose of this study was to determine if the extent of muscle glycogen depletion, muscle damage, and increase in serum cortisol predicted some of the variance measured in exercise-induced increases in plasma and muscle IL-6, IL-8, and MCP-1. Contrary to our hypothesis, change in muscle glycogen was not related to increases in muscle IL-6, IL-8, and MCP-1 mRNA and protein levels, or plasma IL-8 and MCP-1. A modest correlation was measured between changes in muscle glycogen and plasma IL-6, in agreement with our previous, similar study in runners (Nieman et al., 2015). Exercise-induced elevations in the muscle damage biomarker serum myoglobin and serum cortisol both correlated with increases in plasma levels of two chemotactic cytokines, IL-8 and MCP-1, with additional linkages to muscle MCP-1 and IL-8 protein content, respectively.

A strength of this study was the simultaneous measurement of muscle mRNA expression, muscle protein content, and plasma levels for three cytokines (IL-6, IL-8, and MCP-1) after exhaustive exercise (Nieman et al., 2015). Della Gatta et al. (2014) reported that muscle mRNA expression and protein content for IL-6, IL-8, and MCP-1 were increased 2 h following intensive leg resistance exercise. Data also supported the novel finding that exercise-induced cytokine release may have originated from cells in the interstitial space between muscle fibers, including macrophages, satellite cells, and other stromal cells. In the current study, the magnitude of increase in muscle IL-6, IL-8, and MCP-1 mRNA expression and protein content was substantially higher in the cyclists after 75-km cycling (168 ± 26.0 min, 69.6 ± 10.3% VO_2max_) compared to our prior study with runners following a treadmill run to exhaustion (144 ± 6.5 min, 69.2 ± 6.4% VO_2max_) (Nieman et al., 2015). The increase in plasma IL-6, IL-8, and MCP-1, however, was very similar between these two studies. These data from studies conducted in our Human Performance Laboratory with cyclists and runners raise several interesting issues and observations.

Obviously cycling and running have very different metabolic demands on the vastus lateralis muscle, and biopsy samples from this single muscle site provide data with certain limitations when comparing exercise modes and corresponding cytokine responses. There is growing support that multiple cells sources from within skeletal muscle tissue, adipose tissue, and other tissue areas produce cytokines in response to exercise workload challenges (Peake et al., 2015). Thus, a limitation in the current study was the lack of fat biopsy samples that would have improved interpretation of the muscle and plasma data. Endothelial cells, pericytes, fibroblasts, neutrophils, and monocytes/macrophages may all contribute to global cytokine expression within multiple tissue areas of the human body during prolonged and intensive exercise. In addition to skeletal muscle, IL-6 is also released from the brain (Nybo et al., 2002), peritendinous tissue (Langberg et al., 2002), and fat tissue (Keller et al., 2003) during exercise. The net effect, whether the athlete is engaging in long duration running or cycling, is an increase in plasma IL-6, IL-8, MCP-1, and other cytokines that assist physiological, metabolic, and immune processes within the body to cope with the physiological stress (Pedersen, 2013; Catoire and Kersten, 2015; Peake et al., 2015).

In this study in cyclists, and our previous study in runners, we have been unable to link the wide variance in exercise-induced glycogen depletion with cytokine mRNA expression in skeletal muscle tissue. Our data indicate that vastus lateralis muscle glycogen depletion was more than twice as great in the cycling compared to running study, and in both studies was unrelated to muscle IL-6, IL-8, and MCP-1 mRNA expression (Nieman et al., 2015). Plasma IL-6, IL-8, and MCP-1 levels were negatively related to post-run glycogen levels in the running study, with this relationship seen solely for plasma IL-6 in the cycling study. Together these data indicate that muscle glycogen depletion at best represents just one potential signal for cytokine mRNA expression and production, and that other factors have the potential to influence this relationship. For example, we have previously shown that carbohydrate compared to placebo ingestion during prolonged and intensive exercise is related to elevated blood glucose levels, lower serum cortisol and plasma epinephrine concentrations, and decreased muscle mRNA expression and plasma levels for IL-6 and IL-8 despite similar muscle glycogen depletion rates (Nieman et al., 2003, 2005). Others have shown that IL-6 release is markedly higher from the arm compared with the leg during whole-body exercise, and is not linked to muscle glycogen content (Helge et al., 2011). In general, when endurance athletes engage in prolonged, intensive exercise with normal pre-exercise glycogen levels, depletion in muscle glycogen stores varies widely between and typically cannot be linked to muscle IL-6, IL-8, or MCP-1 mRNA expression, with some linkage to increases in plasma cytokine levels (Nieman et al., 2003, 2005, 2015; Holmes et al., 2004; Gusba et al., 2008; Helge et al., 2011).

This study included pre- and post-exercise serum myoglobin, a rapid indicator of exercise-induced muscle damage. The mountainous 75-km cycling course caused a significant increase in serum myoglobin concentrations, but this varied widely between the athletes (53–972 ng/ml). We showed that changes in myoglobin were related to increases in plasma levels of two chemotactic cytokines, IL-8 and MCP-1, and muscle MCP-1 concentrations. At the 160-km Western States Endurance Run (WSER), we reported a strong relationship between the muscle damage biomarker creatine kinase (CK), delayed onset of muscle soreness (DOMS), and a spectrum of systemic inflammation markers including IL-6, IL-8, MCP-1, C-reactive protein (CRP), granulocyte colony stimulating factor (GCSF), IL-1 receptor antagonist (IL-1ra), and IL-10 (Nieman et al., 2006, 2007; Nieman, 2009). During the week following the WSER, DOMS also showed modest positive correlations with many of the cytokines changes experienced during the race (Nieman, 2009). We also reported that muscle damage, muscle soreness, and plasma cytokine levels were linked in a study using a 3-day functional overreaching model (Nieman et al., 2014). Other studies provide variable support for the linkage between muscle damage and plasma cytokine levels, and this may be related in part to the employment of exercise protocols that did not combine the duration and intensity needed to induce sizeable increases in outcome measures (Nieman et al., 2005; Peake et al., 2005; Suzuki et al., 2006; Paulsen et al., 2012; Sugama et al., 2012). The control of cytokine production in skeletal muscle during exercise is complex and likely depends on interactions between a wide variety of local and systemic factors (Peake et al., 2015).

In summary, this study evaluated relationships of three physiologic stress indicators (muscle glycogen depletion, acute muscle damage, elevated levels of the stress hormone cortisol) with increases of three cytokines (IL-6, IL-8, and MCP-1) in two matrixes (muscle and plasma) measured in three ways (muscle mRNA expression and protein content, plasma concentrations) in athletes before and immediately after prolonged and intensive exercise (75-km cycling on an intensive, mountainous course). Although this study design is unique, additional blood and muscle samples collected during recovery may have added valuable data. However, this limitation must be balanced against the rapid resolution of post-exercise plasma cytokine levels to pre-exercise levels. Within this context, the data indicated that increases in muscle IL-6, IL-8, and MCP-1 mRNA after 75-km cycling were unrelated to glycogen depletion and muscle damage. Modest relationships were seen between change in muscle glycogen and increases in plasma IL-6, and changes in serum myoglobin and cortisol to the chemotactic cytokines IL-8 and MCP-1.

In general, this study indicates that muscle glycogen depletion, muscle damage, and increases in serum cortisol during intensive and prolonged cycling are weak predictors of the strong but highly variable post-exercise increase in plasma and muscle IL-6, IL-8, and MCP-1 concentrations. In other words, 75-km of cycling is a major “trigger” for muscle and plasma IL-6, IL-8, and MCP-1 increases, but individual athletes vary widely for reasons that are not explained by the extent of muscle glycogen depletion, muscle damage, and increase in serum cortisol. In practical terms, some athletes performing a 75-km cycling time trial may experience high levels of inflammation compared to others, and future research is needed to discover what best predicts this variance in inflammation and potential influences on recovery.

## Author contributions

DN was the primary investigator and organized all aspects of the study, and wrote the paper. KZ helped devise the study design, collected data, interpreted results, wrote parts of the paper, and edited the paper. DL helped devise the study design, collected data, interpreted results, wrote parts of the paper, and edited the paper. MM helped devise the study design, collected data, interpreted results, wrote parts of the paper, and edited the paper. RH helped devise the study design, collected data, interpreted results, wrote parts of the paper, and edited the paper.

### Conflict of interest statement

The authors declare that the research was conducted in the absence of any commercial or financial relationships that could be construed as a potential conflict of interest.
